# Global longitudinal active strain energy density (GLASED): age and sex differences between young and veteran athletes

**DOI:** 10.1186/s44156-024-00052-1

**Published:** 2024-07-15

**Authors:** David H. MacIver, Henggui Zhang, Christopher Johnson, Efstathios Papatheodorou, Gemma Parry-Williams, Sanjay Sharma, David Oxborough

**Affiliations:** 1 Biological Physics Group, Department of Astronomy and Physics, University of Manchester, Manchester, UK; 2 Department of Cardiology, Taunton & Somerset Hospital, Musgrove Park, UK; 3 Research Institute for Sports and Exercise Science, Liverpool John Moores University, Liverpool, UK; 4 Onassis Cardiac Surgery Centre, Unit of Rare and Inherited Cardiac Disease, Athens, Greece; 5 St George’s University Hospital, London, UK

**Keywords:** Contractance, Contractility, Contractile function, Energy, Heart failure, Systolic function, Stroke work

## Abstract

**Background:**

Global longitudinal active strain energy density (GLASED) is an innovative method for assessing myocardial function and quantifies the work performed per unit volume of the left ventricular myocardium. The GLASED, measured using MRI, is the best prognostic marker currently available. This study aimed to evaluate the feasibility of measuring the GLASED using echocardiography and to investigate potential differences in the GLASED among athletes based on age and sex.

**Methods:**

An echocardiographic study was conducted with male controls, male and female young athletes, and male and female veteran athletes. GLASED was calculated from the myocardial stress and strain.

**Results:**

The mean age (in years) of the young athletes was 21.6 for males and 21.4 for females, while the mean age of the veteran athletes was 53.5 for males and 54.2 for females. GLASED was found to be highest in young male athletes (2.40 kJ/m^3^) and lowest in female veterans (1.96 kJ/m^3^). Veteran males exhibited lower values (1.96 kJ/m3) than young male athletes did (*P* < 0.001). Young females demonstrated greater GLASED (2.28 kJ/m^3^) than did veteran females (*P* < 0.01). However, no significant difference in the GLASED was observed between male and female veterans.

**Conclusion:**

Our findings demonstrated the feasibility of measuring GLASED using echocardiography. GLASED values were greater in young male athletes than in female athletes and decreased with age, suggesting possible physiological differences in their myocardium. The sex-related differences observed in GLASED values among young athletes were no longer present in veteran athletes. We postulate that measuring the GLASED may serve as a useful additional screening tool for cardiac diseases in athletes, particularly for those with borderline phenotypes of hypertrophic and dilated cardiomyopathies.

**Supplementary Information:**

The online version contains supplementary material available at 10.1186/s44156-024-00052-1.

## Background

Long-term intense exercise induces changes in the left ventricles of athletes, including increases in wall thickness and ventricular volumes. These geometric changes are influenced by factors such as age, sex, training duration, sport type, and genetics [1, 2]. Although the left ventricular ejection fraction (LVEF) has been the main measure of systolic function for more than 50 years, recent studies have questioned the reliability of the LVEF due to the impact of structural changes. Specifically, the LVEF is increased by an increase in wall thickness [3, 4] or a decrease in internal diameter [5] and length [6], independent of any change in myocardial strain. These modelling findings have recently been corroborated by other groups [7, 8] and in clinical studies [1, 6, 8]. 

The influence of each structural change on the LVEF can be represented by parabolic curves described using quadratic functions (Appendix Sect. 1). For example, with a 1 mm increase in end-diastolic wall thickness (EDWT), the LVEF increases by between 2.1 and 2.6% points. Conversely, an increase in the left ventricular internal diameter in diastole (LVIDd) of 1 mm decreases the LVEF by 0.4 to 1.2% points. The corrected ejection fraction (EF_c_) was developed to account for the effects of geometric differences and expose the misleading nature of the LVEF [6].

Myocardial strain has been used to address the limitations of LVEF. Increasing myocardial strain magnitude increases LVEF in a curvilinear manner [3]. Moreover, midwall circumferential strain had a greater contribution to the LVEF (2/3) than did long-axis shortening (1/3) [9]. A change in midwall circumferential strain can alter LVEF by 2–3% points for every 1% change in strain (Appendix Sect. 1). Myocardial strain is often reduced in thicker walled ventricles, frequently with preservation of the LVEF [10, 11] due to maintenance of absolute wall thickening [6]. However, myocardial strain is notably affected by afterload [12], limiting its usefulness.

We recently reported a new method for assessing myocardial contractile function called contractance [12]. Contractance is defined and quantified by the myocardial active strain energy density (MASED). The MASED evaluates the mechanical work (energy) performed per unit volume of myocardial tissue during systole [12, 13]. The MASED overcomes the weaknesses of both LVEF and myocardial strain by combining information from both the stress (contractile force per unit cross-sectional area) and myocardial strain. The MASED allows for loading conditions and can be applied in both in vitro/ex vivo [12] and in vivo studies [13, 14], improving the ability to compare research studies. In the left ventricle, contractance can be estimated in both the longitudinal and circumferential directions with the global longitudinal active strain energy density (GLASED) and circumferential active strain energy density (CASED), respectively [13]. We found that GLASED provided a more reliable assessment of contractance than CASED did in a previous study using CMR [13].

The total work done or mechanical energy generated by muscle mass in the longitudinal and circumferential directions are called the global longitudinal active strain energy (GLASE) and circumferential active strain energy (CASE), respectively [13]. GLASE and CASE are similar to stroke work but are derived from myocardial mechanics (stress and strain) rather than from luminal information (stroke volume and pressure).

Using cardiac magnetic resonance (CMR) imaging, we recently assessed GLASE, CASE, GLASED and CASED in cohorts with severe hypertension, dilated cardiomyopathy and amyloid heart disease [13]. We showed that, compared with other left ventricular structural and functional markers, the GLASED was the best method for predicting expected mortality. GLASED is more accurate than LVEF, corrected LVEF, strains, stresses, forces, stroke work, myocardial contraction fraction and pressure strain loops in predicting the expected outcome in patients with dilated cardiomyopathy and amyloid heart disease. Furthermore, GLASED is sensitive enough to detect changes in hypertensive cardiomyopathy [13]. 

GLASED may be better than CASED because circumferential strain varies between ~-3% and ~-35% in the subendocardium and subepicardium, respectively [9]. Furthermore, there is also a large circumferential stress gradient across the wall with high subendocardial and low subepicardial values. The combination of heterogeneous stresses and strains across the wall may have resulted in less reliable CASED values. In contrast, the longitudinal stresses and strains are more homogenous, resulting in more consistent GLASED results [13]. 

In a second study, in a low-risk community-based cohort comprising 44,957 individuals who underwent CMR, GLASED had the highest proportional hazard ratio (HR) for major adverse cardiovascular events and mortality compared with strain, LVEF and 21 other previously proposed structural and functional markers [14]. These two studies showed that the GLASED is the best left ventricular prognostic marker to date.

Given the emerging prognostic data available from the CMR studies described above, our main aim was to determine whether GLASED could also be reliably assessed with echocardiography. We chose populations of athletes of different ages and sexes for whom the relevant echocardiographic data required for the calculation of the GLASED were available. Our prespecified null hypothesis was that there would be no difference in GLASED between the sexes and age groups. Hence, in this retrospective observational cross-sectional study, we sought to assess GLASED in young and veteran male and female athletes combined with a young male nonathlete control group to explore potential differences based on age and sex.

## Methods

### Study population and design

A retrospective analysis of 447 healthy individuals was performed; the participants consisted of 5 cohorts, 245 young male athletes (mixed sports), 67 young female athletes (soccer), 70 veteran male athletes (mixed sports), 44 veteran female athletes (mixed sports) and 21 healthy nonathletes. Data from one of these cohorts has been published [1]. The data were collected either as part of the mandatory preparticipation cardiac screening or as part of a planned and structured research study. Most of the healthy controls participated in recreational exercise/activity. Participants completed a health screening questionnaire to determine the presence of any cardiovascular symptoms, family history of sudden cardiac death or other cardiovascular history. Blood pressure was recorded using a standard sphygmomanometer. A resting 12-lead ECG and transthoracic echocardiogram were performed on all participants. A sports cardiologist (SS) reviewed all the results. No individuals were excluded because of imaging or clinical reasons. Ethics approval was obtained from the ethics committees of Liverpool John Moores University and St Georges University Hospital.

### Echocardiography

A standard echocardiogram was performed by experienced sonographers accredited by the British Society of Echocardiography (BSE) using a commercially available ultrasound system (Vivid Q or Vivid E95, GE Healthcare, Horten, Norway) with a 1.5-4 MHz phased array transducer, with the participant lying in the left lateral decubitus position. All images were obtained in accordance with the BSE guidelines [15]. The images were stored in a raw digital imaging and communications in medicine (DICOM) format and exported to an offline analysis system (EchoPac version 202, GE Healthcare, Horton, Norway) for subsequent analysis. The mean E’ velocities were calculated from the mean of the medial and lateral values using tissue Doppler. Doppler studies were not performed for the veteran male cohort.

The parasternal short-axis orientation was used to measure the end-diastolic wall thickness (EDWT) and left ventricular end-diastolic internal diameter (LVIDd). Measurements were obtained at the basal and mid-levels from the anteroseptum, inferoseptum, inferior, posterior, lateral and anterior walls. The basal short axis was located at the tip of the MV, and the mid-short axis was located at the papillary muscle level. Each segment was measured once at each basal and mid-level in each of the 6 segments. The mean EDWT and mean LVIDd were calculated from the 12 wall thicknesses and 6 LVIDd dimensions.

Integrated myocardial speckle tracking software was used to calculate longitudinal and circumferential strain. Apical 4-, 3- and 2-chamber orientations were used to derive global longitudinal strain (GLS), while the parasternal short axis at the basal, mid- and apical levels was acquired for global circumferential strain. Images were optimised to maximise endocardial delineation, and frame rates were maintained between 40 and 90 fps. Offline analysis allowed for semiautomated tracking, and all images had acceptable tracking of all segments. Reproducibility for measuring strain has previously been reported to be good in our laboratory using a repeated measures acquisition study [16]. Circumferential strains were not available for young female athletes. Left ventricular muscle mass was calculated using the regression equation recommended by Lang and colleagues [17]. 

### Global longitudinal active strain energy density

The peak longitudinal stress was calculated using the Lamé equation. The Lamé equations were used for calculating nominal stresses, as the Laplace method is accurate only for thin-walled chambers with a diameter/thickness < 20 [13, 18], as follows:

$${\rm{Longitudinal}}\,{\rm{Lam \acute{e} }}\,{\rm{stress}} ({{\sigma }}_{\text{l}})=\frac{{\text{P}}_{\text{i}}{{\text{r}}_{\text{i}}}^{2} }{({{\text{r}}_{\text{o}}}^{2}-{{\text{r}}_{\text{i}}}^{2})}$$

where P_i_ is the inner (ventricular cavity) pressure (in Pa) and is equal to the peak systolic pressure. A brachial cuff derived from systolic blood pressure was used for P_i_. Furthermore, r_o_ is the outer (epicardial), and r_i_ is the inner (luminal or endocardial) LV radii, respectively.

The nominal longitudinal force was calculated from the product of the longitudinal Lamé stress and the end-diastolic short-axis cross-sectional myocardial area.

GLASED was calculated using the following equation: [13, 14]

$$\text{G}\text{L}\text{A}\text{S}\text{E}\text{D}=\frac{1}{2} \times {\sigma }_{l}\times \left|{\epsilon}_{l}\right|$$

where ${\sigma }_{l}$ is the longitudinal nominal stress (contractile stress is positive by convention) and $\left|{\epsilon}_{l}\right|$ is the magnitude of the peak longitudinal strain.

GLASE was calculated by multiplying GLASED by the LV muscle volume derived from the muscle mass using the equation above and assuming a myocardial density of 1.05 g/ml [17]. To account for body size, GLASE was indexed to BSA [17] and height^2.7^ [19]. 

Global longitudinal power (GLP) was calculated using the following equation:

$$\text{G}\text{L}\text{P}=\text{G}\text{L}\text{A}\text{S}\text{E} \times \text{h}\text{e}\text{a}\text{r}\text{t} \text{ r}\text{a}\text{t}\text{e} \left(\text{b}\text{p}\text{m}\right)/60$$

### Relationships between LVEF and structural differences

A mathematical modelling substudy was performed to assess the impact of differences in left ventricular geometry and strain on LVEF to clarify the differences in LVEF found in our study of athletes. The method has been described in detail elsewhere [1, 6]. The two-shell model was used, and the left ventricular end-diastolic diameter, wall thickness and strain were altered to assess their effect on LVEF. Changes in LVEF were obtained by individually adjusting the following input variables: midwall circumferential shortening 15–20%, EDWT 10 to 15 mm and LVIDd 40–50 mm. Quadratic equations were derived from the resulting curves, allowing the relative impact of each variable to be calculated (see Appendix 1). The equations obtained were subsequently used to calculate the expected differences in LVEF from the relevant input variables from the cohorts and compared with the measured LVEF.

### Statistical analysis

Our primary a priori null hypothesis was that there was no significant difference in GLASED between sexes in the different age groups. Normality was assessed using the Shapiro‒Wilk test. Either two-tailed t tests or Mann‒Whitney tests were performed on these pairs as appropriate. To allow for multiple comparisons, one-way analysis of variance (ANOVA) was performed for all comparisons with Tukey HSD/KRAMER analysis when both cohorts had a normal distribution, and the Kruskal‒Wallis test/Nemenyi test was used when either cohort was not normally distributed. Correlations were assessed using Pearson’s method.

## Results

### Demographic and echocardiographic findings

No individuals had clinically significant valvular disease or any overt cardiomyopathic processes. All the results and their statistical significance are shown in Table 1. There was no significant difference in age between young male and young female athletes (21.6 and 21.4 years, respectively; ns). Veteran males and females were similar in age (53.5 vs. 54.2 years, ns).

**Table 1:** Demographics and results (mean ± 1 SD)

	All	Control male	Young male	Young female	Veteran males	Veteran females	Significance
Number	447	21	245	67	70	44	
Age (years)	29.8 ± 15.0	21.2 ± 1.09	21.6 ± 5.2	21.4 ± 4.3	53.5 ± 6.6	54.2 ± 6.0	ns, ns,‡‡‡,§§§,ns
Height (m)	1.76 ± 0.12	1.79 ± 0.08	1.81 ± 0.07	1.66 ± 0.05	1.78 ± 0.07	1.66 ± 0.07	ns,†††,‡‡‡,ns,¶¶¶
Training duration (hours/week)	13.7 ± 7.6	2.8 ± 4.0	16.9 ± 7.9	13.1 ± 4.3	8.7 ± 2.6	9.2 ± 3.7	***,†,‡‡‡,ns, ns
Years of training (years)	18.1 ± 11.1	8.3 ± 5.7	13.4 ± 4.6	13.9 ± 4.4	31.8 ± 13.7	30.8 ± 10.7	ns, ns,‡‡‡,§§§,ns
Height^2.7^ (m^2.7^)	4.67 ± 0.64	4.84 ± 0.49	4.96 ± 0.49	3.94 ± 0.35	4.78 ± 0.54	3.95 ± 0.45	ns,†††,‡‡‡,ns,¶¶¶
Weight (kg)	88.9 ± 13.9	75.8 ± 11.8	88.9 ± 13.9	63.2 ± 7.1	76.0 ± 9.1	59.1 ± 7.9	**,†††,‡‡‡,ns,¶¶¶
BMI (kg/m^2^)	25.2 ± 3.6	23.6 ± 3.1	27.1 ± 3.3	22.9 ± 2.2	23.9 ± 2.3	21.4 ± 2.1	***,†††,‡‡‡,ns,¶¶¶
BSA (m^2^)	1.96 ± 0.23	1.94 ± 0.16	2.09 ± 0.18	1.70 ± 0.11	1.94 ± 0.14	1.65 ± 0.13	*,†††,‡‡‡,ns,¶¶¶
Heart rate (bpm)	57.5 ± 10.4	70.9 ± 10.7	56.6 ± 9.5	58.5 ± 10.2	55.3 ± 10.4	58.1 ± 11.1	***,ns, ns, ns, ns
Systolic BP (mmHg)	127 ± 12	129 ± 10	130 ± 9	117 ± 13	130 ± 15	116 ± 12	ns,†††,ns, ns,¶¶¶
Diastolic BP (mmHg)	71.1 ± 8.5	74.4 ± 7.0	68.7 ± 6.6	67.2 ± 8.01	79.9 ± 7.8	74.9 ± 7.2	*,ns,‡‡‡,§§§,ns
LV muscle mass (g)	160 ± 41	121 ± 21	178 ± 32	119 ± 20	174 ± 45	120 ± 28	***,†††,ns, ns,¶¶¶
LVIDd (mm)	53.9 ± 4.44	48.8 ± 3.7	54.8 ± 3.8	49.2 ± 3.4	52.0 ± 4.8	46.0 ± 3.6	***,†††,‡‡‡,§§§,¶¶¶
EDWT (mm)	8.42 ± 1.07	7.53 ± 0.47	8.68 ± 0.8	7.32 ± 0.61	9.10 ± 1.26	8.03 ± 1.30	***,†††,‡‡,§§§,¶¶¶
LVIDs (mm)	35.1 ± 4.6	34.4 ± 3.0	37.1 ± 3.7	32.3 ± 3.4	34.2 ± 5.9	30.2 ± 3.0	***,†††,‡‡‡,ns,¶¶¶
LVEF (%)	60.1 ± 5.3	59.4 ± 3.6	58.9 ± 4.7	61.7 ± 5.1	59.9 ± 5.9	65.8 ± 5.3	ns,†††,ns,§§§,ns
Mean S’ (cm/s)	9.9 ± 2.0	11.1 ± 2.0	10.3 ± 1.6	9.9 ± 1.5	8.6 ± 3.0	8.2 ± 1.3	ns, ns,‡‡‡,§§§,ns
Mean E/A ratio	2.07 ± 0.71	1.73 ± 0.45	2.19 ± 0.65	2.41 ± 0.79	dna	1.26 ± 0.42	***,ns, dna,§§§,dna
Mean E’ (cm/s)	15.8 ± 2.94	16.4 ± 3.02	16.2 ± 2.42	16.8 ± 2.67	dna	11.4 ± 2.28	ns, ns, dna,§§§,dna
Mean E/E’	5.38 ± 1.13	5.08 ± 1.01	5.31 ± 1.02	5.41 ± 1.21	dna	6.11 ± 1.46	ns, ns, dna,§,dna
GLS (%)	-19.7 ± 2.5	-19.0 ± 1.6	-20.1 ± 2.1	-19.9 ± 2.7	-18.4 ± 2.3	-19.6 ± 2.3	ns, ns, ns, ns, ns
Lamé longitudinal σ SBP (kPa)	22.9 ± 3.8	24.2 ± 3.1	23.8 ± 3.0	23.0 ± 3.3	21.3 ± 4.2	19.6 ± 4.8	ns, ns,‡‡‡,§§§,ns
Peak longitudinal force (N)	37.0 ± 8.5	32.3 ± 5.3	41.2 ± 6.6	30.0 ± 6.6	37.0 ± 8.4	26.1 ± 5.0	***,†††,‡‡,§§§,¶¶¶
Peak longitudinal force/LVM (N/mg)	204 ± 34	237 ± 30	201 ± 26	225 ± 32	188 ± 39	202 ± 51	***,†††,ns,§§,ns
GLASED (kJ/m^3^)	2.27 ± 0.48	2.31 ± 0.39	2.40 ± 0.42	2.28 ± 0.41	1.96 ± 0.51	1.92 ± 0.51	ns,†,‡‡‡,§§,ns
GLASE (mJ)	346 ± 116	268 ± 66	406 ± 98	260 ± 70	320 ± 102	215 ± 58	***,†††,‡‡‡,ns,¶¶¶
GLASE/BSA (mJ/m^2^)	175 ± 49.3	139 ± 34.2	195 ± 44.3	153 ± 38.0	165 ± 51.6	130 ± 34.4	***,†††,‡‡‡,ns,¶¶
GLASE/H^2.7^ (mJ/m^2.7^)	73.6 ± 21.1	56.1 ± 15.7	82.2 ± 19.0	66.1 ± 16.7	67.4 ± 21.2	54.8 ± 15.2	***,†††,‡‡‡,ns,¶
GLP (mW)	327 ± 113	313 ± 79	381 ± 101	250 ± 69	293 ± 104	207 ± 67	***,†††,‡‡‡,ns,¶¶

EDWT, end-diastolic wall thickness; MWT, mean wall thickness; LVIDd, left ventricular internal diastolic diameter; GLS, global longitudinal strain; σ, stress. GLASED, global longitudinal active strain energy density; GLASE, global longitudinal active strain energy; GLP, global longitudinal power. Significance: *control male v young male, †young male v young female, ‡young male v veteran male, §young female v veteran female, ¶veteran male v veteran female. *P < 0.05, ** *P* < 0.01, ***P < 0.001, ns = not significant and dna = data not available

Compared with young female athletes, young male athletes were taller and heavier; had a greater BMI and BSA; and had a greater SBP, LV mass, LVIDd, MWT and EDWT. Veteran males were taller and heavier than veteran females were. There was no significant difference in the mean heart rate between the athletes, but control males had a greater mean heart rate. Compared to female athletes, male athletes had greater systolic blood pressure. Veteran athletes had higher diastolic blood pressures than young athletes. The LVEF was greater in females in both age groups, with the difference being greater in the veteran athletes.

The mitral E/A ratio was greater in young females than in young males and in young females than in veteran females. The mean E’ was the lowest, and the E/E’ ratio was the highest in female veteran athletes. S’ was lower in the veteran athletes, but there were no differences between the sexes (Table 1).

### Myocardial stresses and strains

**Fig. 1:**
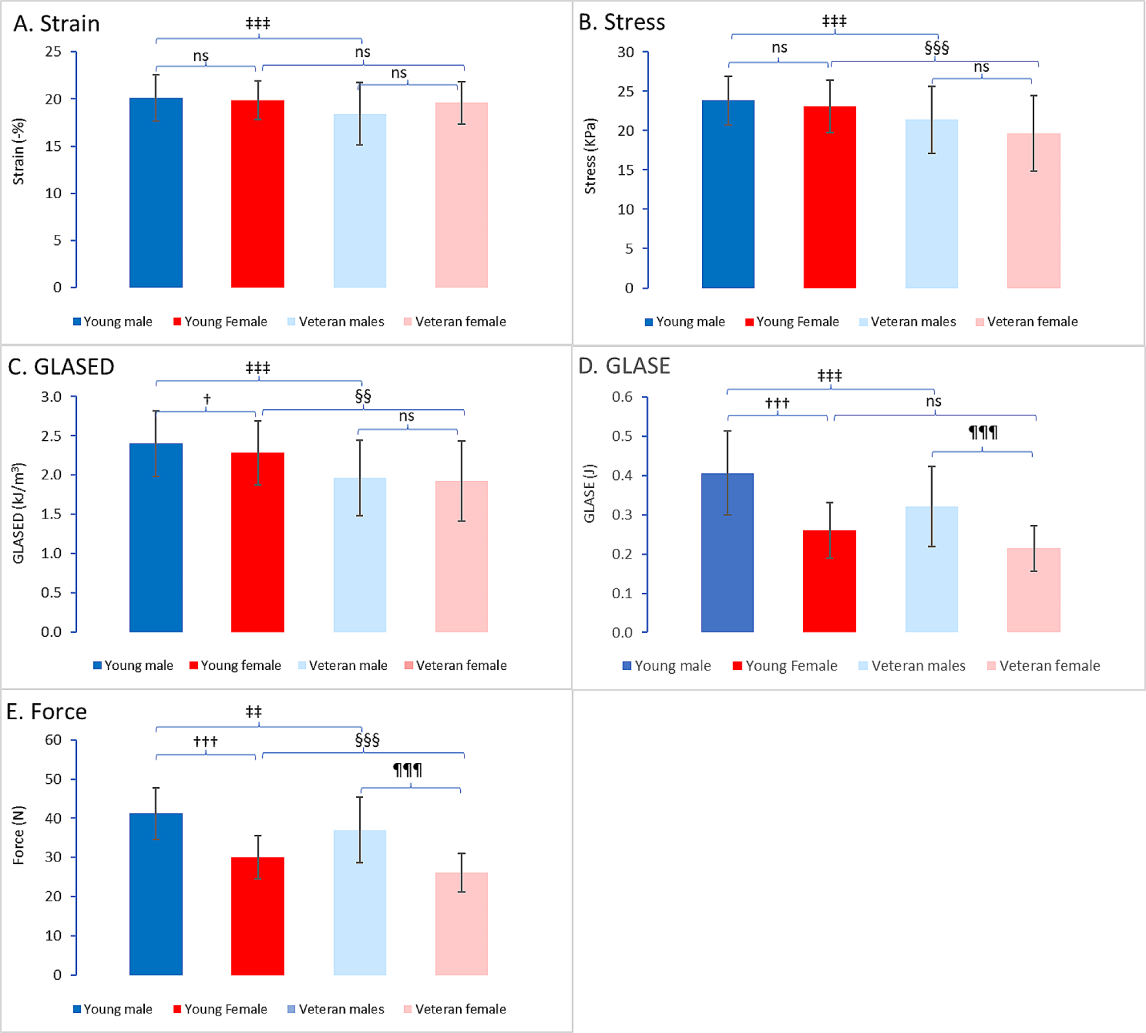
Charts showing the values for global longitudinal strain (**A**), Lamé longitudinal stress (**B**), GLASED (**C**), GLASE (**D**) and peak longitudinal force (**E**)

Global longitudinal strain was not significantly different between males and females or young athletes and veterans (Fig. 1A). Longitudinal contractile wall stress was greater in young male athletes than in veteran athletes, with the greatest decrease occurring in veteran females (Fig. 1B).

### GLASED

**Fig. 2:**
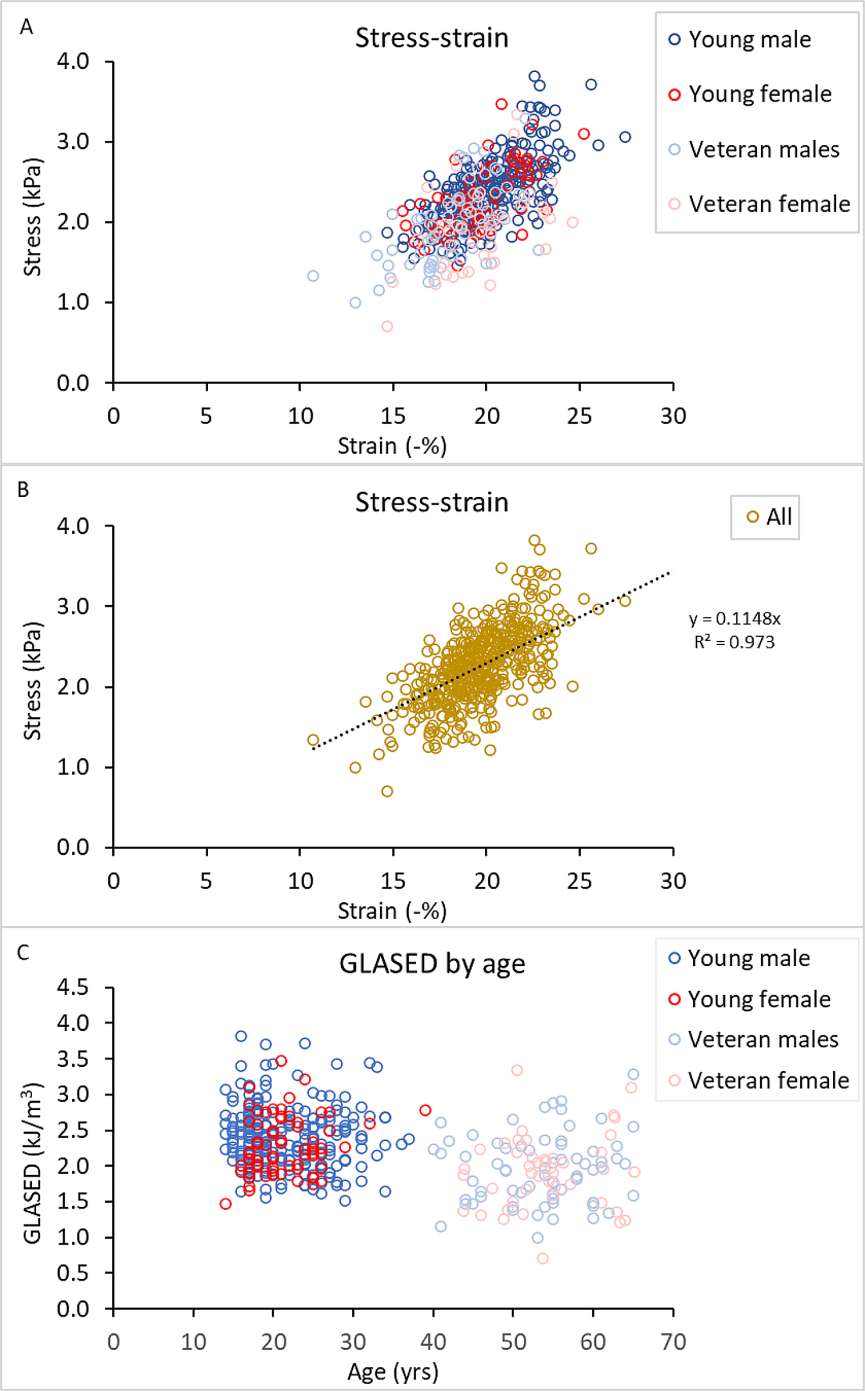
Scatter plots showing (**A**) Relationships between stress and strain according to cohort. (**B**) Stress‒strain relationship for all cohorts and (**C**) relationship between the GLASED score and age

GLASED was calculated for all individuals. The GLASED was highest in young male athletes (2.40 kJ/m^3^) and significantly greater than that in young female athletes (2.28 kJ/m^3^, *P* < 0.05) (Fig. 1C). Compared with their sex-matched counterparts, male and female veterans had a significantly lower GLASED (1.96 and 1.92 kJ/m3, *P* < 0.001 and *P* < 0.01, respectively) (Figs. 1C and 2). Young male nonathletes had a trend toward lower GLASED values (2.27 kJ/m^3^) than young male athletes did (not significant).

### GLASE

Left ventricular myocardial work was assessed using GLASE. GLASE was highest in young male athletes and decreased in veteran male athletes (Fig. 1D). Compared with male patients, female patients had a lower GLASE. The GLASE values of young female athletes were similar to those of veteran female athletes. GLASE was greater in young male athletes than in young male nonathletes (475 vs. 303 mJ, *P* < 0.001).

### Longitudinal forces

There was a significantly greater longitudinal peak force in males than in females, and younger athletes had a greater longitudinal peak force than older athletes did (Fig. 1E).

### Global longitudinal power

**Fig. 3:**
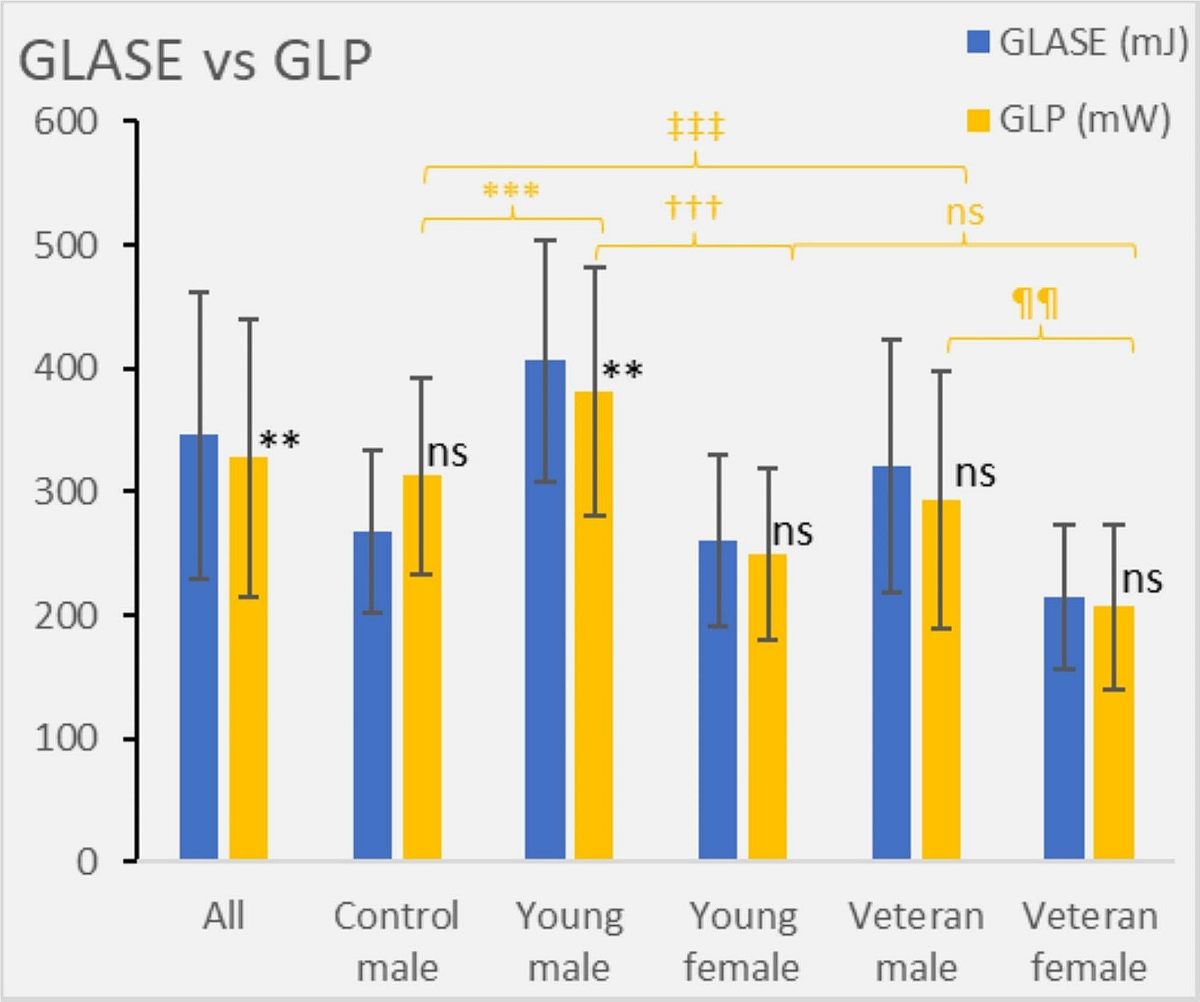
Chart showing global longitudinal active strain energy (work) in comparison to global longitudinal power

Left ventricular longitudinal power was calculated using GLASE and heart rate in a post hoc (exploratory) analysis (Fig. 3). A trend toward greater GLP in male controls was related to their higher heart rate. In contrast, in the athletes, there was a trend toward a lower GLP than the GLASE, which was significant in the young male athletes (*P* = 0.004).

### Relationship between stress and strain

Figure 2A shows the relationship between stress and strain in each cohort. The magnitude of myocardial strain increases (is more negative) as myocardial contractile stress increases. Figure 2B shows the same data for all cohorts, including male controls, and reveals a strong correlation (R^2^ of 0.931) when the intercept was set at 0 and a reasonable correlation (R^2^ of 0.64) when the intercept was not predefined (*P* < 0.0001).

### Relationship between GLASED and age

Figure 2C shows scatter plots of the association between age and GLASED, which revealed lower values in the older age groups.

### Relationships between LVEF and structural differences

The modelling substudy confirmed that changes in LVEF are nonlinear with each individual variable and that the steepness and shape of the slope are dependent on each of the other variables (see Appendix 1 for further details). These figures represent the changes based on an alteration of a single variable with the other variables fixed as follows: LVIDd 45 mm, EDWT 10 mm and magnitude of midwall circumferential shortening of 18.7%.

The substudy showed that the greater LVEF observed in female athletes than in male athletes can be explained by the differences in structure, namely, the lower end-diastolic diameter despite a lower EDWT (Table 2 and Appendix 2).

**Table 2:** Changes in LVEF due to structural differences and strain

Variable	LVEF	Regression coefficient*	Mathematical model**	References
↑GLS	↑↑	+0.65/%	-	[6, 9, 27]
↑mwCS	↑↑↑	+2.1/%	+3.2/%	[6, 9, 27]
↑EDWT	↑↑↑	+2.1/mm	+2.6/mm	[3, 6, 27]
↑LVIDd	↓↓	-0.42/mm	-1.2/mm	[6, 27]
↑LV length	↓	-0.17/mm	-	[6]

GLS, magnitude (absolute value) of global longitudinal strain; mwCS, magnitude of midwall circumferential strain; EDWT, end-diastolic wall thickness; LVIDd, left ventricular internal diameter in diastole; LV length, left ventricular internal long axis length in diastole

*Obtained using a five-variable linear statistical model

**Derived from the 5-variable mathematical model used in [6]

## Discussion

To the best of our knowledge, this is the first echocardiographic study to assess GLASED. The study confirmed that calculating the GLASED using echocardiography was practical and can therefore be readily implemented in clinical practice.

Athletes are known to have altered left ventricular geometry [2], which impacts traditional measures of myocardial systolic function, such as LVEF [1]. Myocardial strain is influenced by afterload [12], and there are differences in blood pressure between the sexes in athletes [20]. The strain energy density is calculated from the stress (i.e., wall thickness, diameter and pressure) and strain and has a long standing background in engineering science. GLASED provides theoretical advantages over established methods, as GLASED corrects for potential confounders such as differences in afterload (e.g., blood pressure) and ventricular remodelling.

We showed significantly greater contractance in young males than in young females and young athletes than in veteran athletes. Potential alternative measures of cardiac function, such as myocardial strain and LVEF, in isolation were unhelpful in distinguishing between the sexes. Plausible explanations for the sex differences in GLASED in young athletes include intrinsic genetic or hormonal differences, type of sports undertaken, training methods and levels of fitness.

The greater GLASED in younger athletes than in veterans indicates that the former produce more mechanical work (energy) per unit volume of myocardium and may indicate that the cardiac muscle is intrinsically stronger for a given unit mass. Nonspecific age-related deterioration, type of sport or training regime, prolonged training damage with myocardial cell death and replacement fibrosis, or hormonal changes could explain the decreased contractance in the veteran cohorts. The lack of a difference between GLASED in young male controls and young male athletes might suggest the absence of a training effect and a widening of its utility.

The LVEF is calculated from ventricular luminal information alone and is influenced by changes in systemic pressure, geometry and strain. It has been previously shown that a larger internal diameter [5] and length decrease the LVEF [6], whereas a greater EDWT increases the LVEF [1, 3]. The combination of higher LVIDd and EDWT has opposite effects on LVEF [3, 5]. Previous studies have also shown a greater LVEF in females than in males [1, 21, 22]. Our finding of a greater LVEF in female young athletes and female veterans is explained by the lower left ventricular diameter and length because a smaller left ventricular diameter and length increase LVEF [1, 6] despite similar strains and a lower wall thickness in females. Substudy modelling [6] predicts that young females would have an LVEF 3.1% greater than that of their male counterparts and that the LVEF of veteran females would be 4.9% greater than that of their male counterparts, which is close to the 3% and 6% we observed (Appendix 2).

The lower resting blood pressure found in female athletes is consistent with previous observations [20]. Neither the GLS nor the GCS was useful for distinguishing between the cohorts. Longitudinal stress was, however, more useful in comparing young and veteran athletes but was of no benefit in assessing differences between the sexes.

The reference ranges (95% confidence intervals) varied across our cohorts, with GLASED values less than approximately 1.5 kJ/m^3^ in young athletes and less than 1.0 and 0.9 kJ/m^3^ in male and female veterans, respectively (Table 3). Such reference ranges may be useful in identifying left ventricular myocardial diseases such as hypertrophic and dilated cardiomyopathies where the phenotype is uncertain using conventional findings.

**Table 3:** The reference range for GLASED in the different cohorts (age range) was mean ± 1.96 × 1 SD

GLASED (kJ/m^3^)	All (14–65)	Male control (20–25)	Male athlete (14–37)	Female athlete (14–39)	Male veteran (40–65)	Female veteran (44–65)
Upper	3.21	3.08	3.22	3.09	2.91	2.93
Lower	1.34	1.54	1.58	1.48	1.02	0.92

The greater GLASE and longitudinal forces in young male athletes than in young male nonathletes indicate that training is affected by an increase in muscle mass. GLASE provides a measure of the work performed by the whole ventricular muscle mass in the longitudinal direction. GLASE differs from stroke work, as the latter is calculated solely using data from within the lumen (i.e., stroke volume and intracavity pressure), while GLASE is calculated with information from the myocardium itself. Stroke work does not allow for any changes in ventricular geometry, such as increases in wall thickness or ventricular size. In contrast, GLASE uses information from the myocardium directly by calculating contractile stresses and inputting myocardial strains and, therefore, has theoretical advantages over stroke work. The higher mean GLASE value in males remained greater despite correcting for body size. The GLASE, although calculated from stress and strain, is mathematically equivalent to the force exerted longitudinally and distance travelled by the myocardium (longitudinal shortening). The variations in GLASE and longitudinal forces between the cohorts may, in part, explain the difference in their expected athlete performances.

In this study, we used a new metric called global longitudinal power for the first time. The GLP was calculated using GLASE and heart rate in a post hoc analysis. The results showed a similar pattern and magnitude to GLASE apart from the male controls, where GLP was higher than GLASE as a consequence of their higher heart rates.

In contrast to GLASE, which measures work done per beat, GLP calculates the work rate measured in Watts. Although this was exploratory, it shows the potential impact of heart rate, particularly in the control group where GLP was greater than GLASE.

A previous study assessing GLASED reported comparable results. In the CMR study [13], the normal cohort had a mean GLASED of 1.94 kJ/m^3^, the hypertensive group with early hypertensive cardiomyopathy had a GLASED of 1.39 kJ/m^3^, the group with dilated cardiomyopathy had a GLASED of 0.86 kJ/m^3,^ and the group with amyloid heart disease had a GLASED of 0.58 kJ/m^3^. These findings are slightly different from those of the GLASED in this echocardiography study because of the diverse types of strains used, with quantitatively different results (15.4% vs. 19.9%). These differences are attributed to the fact that the CMR study was based on long-axis shortening measured directly using engineering (nominal) strain, whereas the echocardiogram employed software-derived speckle tracking that measured global longitudinal strain [18]. In contrast, stress was lower in the combined echocardiogram cohort than in the CMR-derived normal control cohort (22.9 kPa and 25.1 kPa, respectively).

The calculation of GLASED is straightforward using, for example, the spreadsheet available as a supplement online, which provides the calculation of GLASED using the input variables of systolic blood pressure, left ventricular end-diastolic diameter and end-diastolic wall thickness.

### Limitations and future directions

The sizes and different sports of the groups differed; for example, the young females were soccer players, and therefore, the findings may not solely represent changes attributable to sex and age alone. The majority of the controls and all athlete populations included in this study were of Caucasian ethnicity, which limits the generalizability of the findings to other ethnic groups. Future studies are necessary to clarify the specific effects of ethnicity, different sports and training on GLASED and GLASE.

This study utilised GLS measured using software-derived speckle tracking, which may have limitations in terms of accuracy. A precise assessment of contractance is most accurately evaluated using the area under the stress‒strain curve, which is readily applicable to ex vivo studies using trabeculae [12]. However, this numerical integration method is impractical in clinical practice [13]. An approximation of the area under the stress‒strain curve can be made using the more ‘user-friendly’ method derived from the nominal stress. A comparison of GLASED using the simplified equation (analytic method) and longitudinal contractance using numerical integration of the stress‒strain curve is presented in Appendices 3 and 4, confirming that the simplified equation is a reasonable approximation of the contractance calculated numerically.

The temporally fluctuating and highly heterogeneous deviatoric and hydrostatic stresses and strains can be expressed with manifold three-dimensional tensors, and the strain energy density can be calculated from the double dot product of the individual stress and strain tensors (for a linear elastic material $W=\frac{1}{2}\sigma :\epsilon$). There is significant disagreement regarding the values assigned to material properties of the myocardium, such as the elastic modulus, which are essential for precise finite element modelling [23, 24]. The differences in myocardial properties are likely related to the relative health of the tissues undergoing ex vivo analysis and to factors such as tissue hypoxia, perfusion, temperature and the surrounding milieu. Moreover, the myocardium is hyperelastic and anisotropic, leading to sophisticated modelling that is currently under investigation by our group [23, 25]. 

Our cohorts all had normal ECGs, although it is acknowledged that mild dyssynchrony can be present in athletes, particularly when the ejection fraction is less than 52% [26]. Given that GLASED uses average stress and strain, it is unlikely that mild dyssynchrony will have affected the results.

Despite these limitations, the uniaxial approach presented in this study is simple, easy to apply, and, importantly, scalable with echocardiography. Further details supporting the use of nominal rather than instantaneous stress are provided in the Appendix (Sect. 3 and 4). Although the estimations of strain energy density may not be perfect using GLASED, they are precise enough for clinical use since GLASED performs better than both strain and LVEF in predicting outcome [13, 14] while also reducing the risk of computational error compared with the numerical method for estimating contractance.

Invasive left ventricular pressures could be used for calculating wall stresses more accurately but were not measured because this approach is not realistic in clinical practice. Therefore, the peak systolic pressure derived noninvasively using a brachial cuff was used as a surrogate. We acknowledge that measuring pressure using a sphygmomanometer may influence the calculation of wall stress. Ambulatory BP measurements may further improve the accuracy of SBP measurements. Circumferential strain data were not collected for the young female group, which prevented the calculation of the CASED. We did not have a female control group available, nor did we directly assess the impact of distinct types of sports on the GLASED, which may have influenced our results. Exercise treadmill testing using VO_2 max_ was not available for these cohorts, so the influence of relative fitness could not be assessed.

Stroke work was not assessed because stroke volume data were not available. Based on our previous MRI study [13, 14], stroke work was found to be unhelpful in predicting expected mortality. We provided information on myocardial work using GLASE. We suggest that work calculated from luminal data alone (i.e., stroke work) should be improved by incorporating information derived from myocardial mechanics (combining stress and strain) using contractance. Regional changes and dyssynchrony were not assessed; however, we do not expect significant regional abnormalities given the healthy cohorts with normal ECGs in this study.

Propagation errors arise when the input variables, such as LVIDd, are squared in the GLASED equation. Therefore, the accuracy of such measurements is crucial for obtaining dependable contractance values. Although we have provided reference ranges for each cohort, we acknowledge that our sample sizes were limited and may not be comparable to those of studies performed on echocardiographic equipment from different vendors. Nonetheless, we posit that the reference ranges may be helpful in assessing myocardial function in situations where cardiomyopathic processes are suspected and other measures are inconclusive, as our previous work has shown clinical utility in disease processes [13, 14]. 

## Conclusions

This observational study used echocardiography to assess a novel measure of myocardial contractile function called GLASED. GLASED is easy to calculate using the template provided online and is obtained using only 4 pieces of information, namely, mean LV wall thickness, mean LV diameter, systolic pressure and GLS. Our findings revealed that young male athletes exhibit higher GLASED values than young female athletes do, and the GLASED decreases with age, while the sex differences observed in young athletes disappear among veteran athletes. Additionally, in our substudy, we explain why there are differences in left ventricular ejection fraction (LVEF) between the sexes.

The results of our study hold significant clinical relevance, as they shed light on myocardial function and its potential implications in the screening of cardiac diseases, including dilated and hypertrophic cardiomyopathies. Specifically, a GLASED value below the reference range may indicate reduced energy production per unit volume of muscle, suggesting the occurrence of a cardiomyopathic process. This highlights the importance of further investigating the clinical utility of the GLASED as a tool for evaluating myocardial function and prognosis in individuals with cardiac disorders.

Our research opens new avenues for understanding and monitoring myocardial (dys)function. As such, these findings may contribute to enhanced diagnostic accuracy and improved management of cardiac diseases, particularly in the context of athlete screening and evaluation of borderline phenotypes.

To fully determine the potential benefits of GLASED in clinical practice, future studies should further explore its predictive ability and establish standardised reference ranges. We speculate that the inclusion of the GLASED as part of a comprehensive cardiac assessment may lead to improved patient outcomes and better-informed treatment decisions in individuals at risk of or diagnosed with cardiac disorders.

### Electronic supplementary material

Below is the link to the electronic supplementary material.

Supplementary Material 1

## Data Availability

No datasets were generated or analysed during the current study.

## List of abbreviations

CASED: Circumferential active strain energy density
GLASED: Global longitudinal active strain energy density
GLP: Global longitudinal power
