# Supplementary material for: Global longitudinal active strain energy density (GLASED): age and sex differences between young and veteran athletes
Source: Echo Res Pract. 2024 Jul 15;11:17. doi: 10.1186/s44156-024-00052-1 (PMC11247749; doi:10.1186/s44156-024-00052-1)

**Appendix (online supplement)**

*1. How do LVIDd, wall thickness and myocardial strain impact LVEF?*


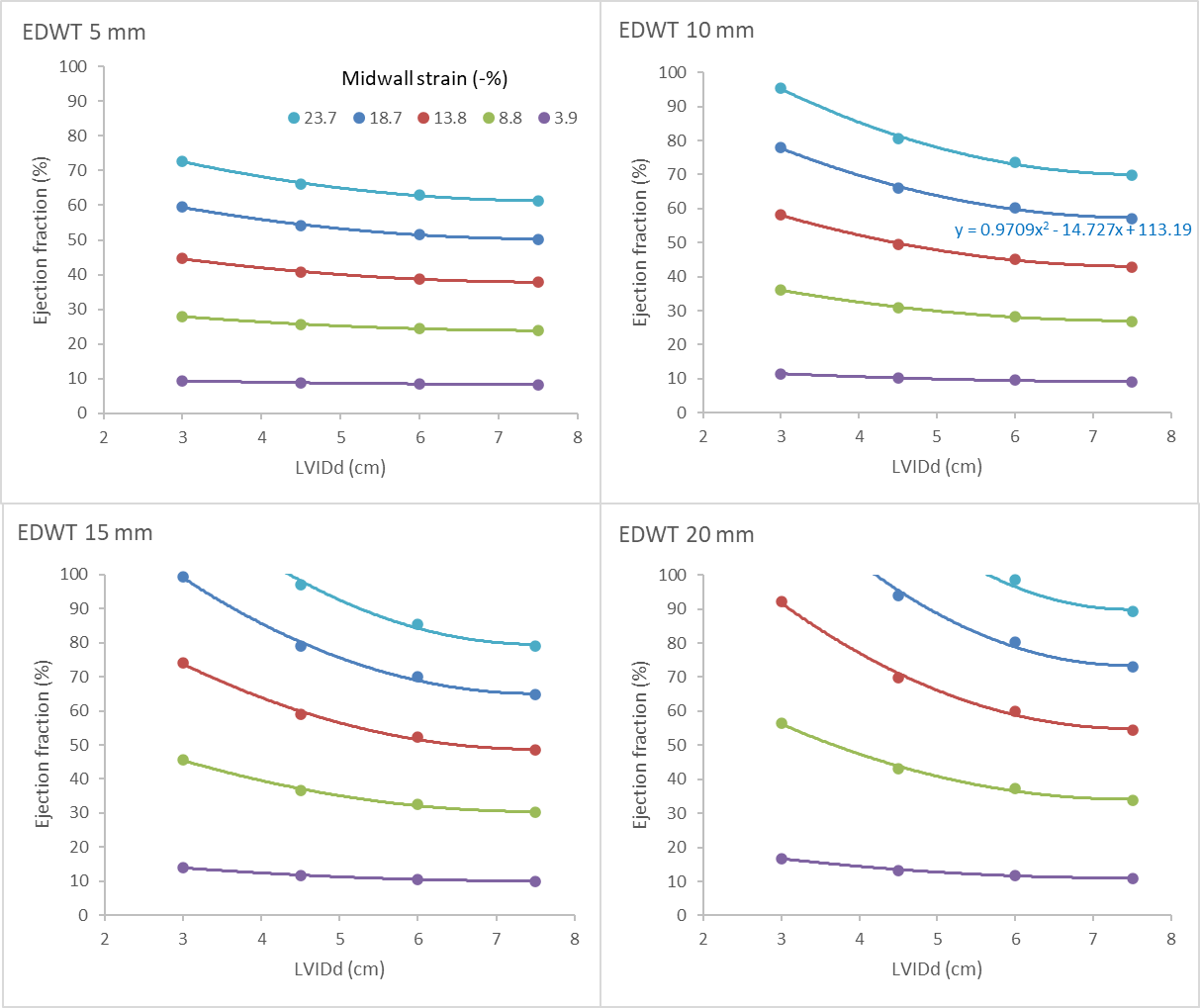


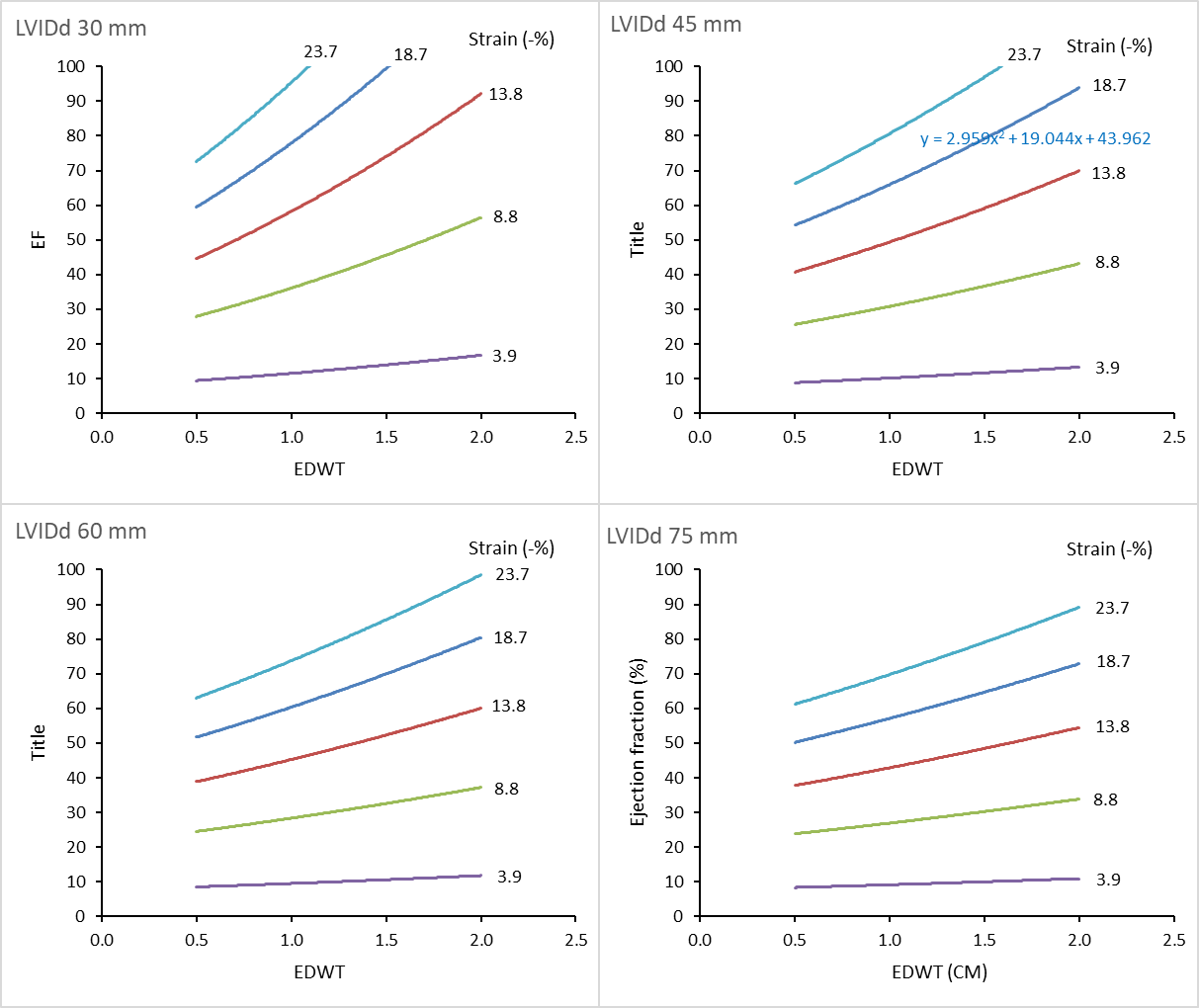


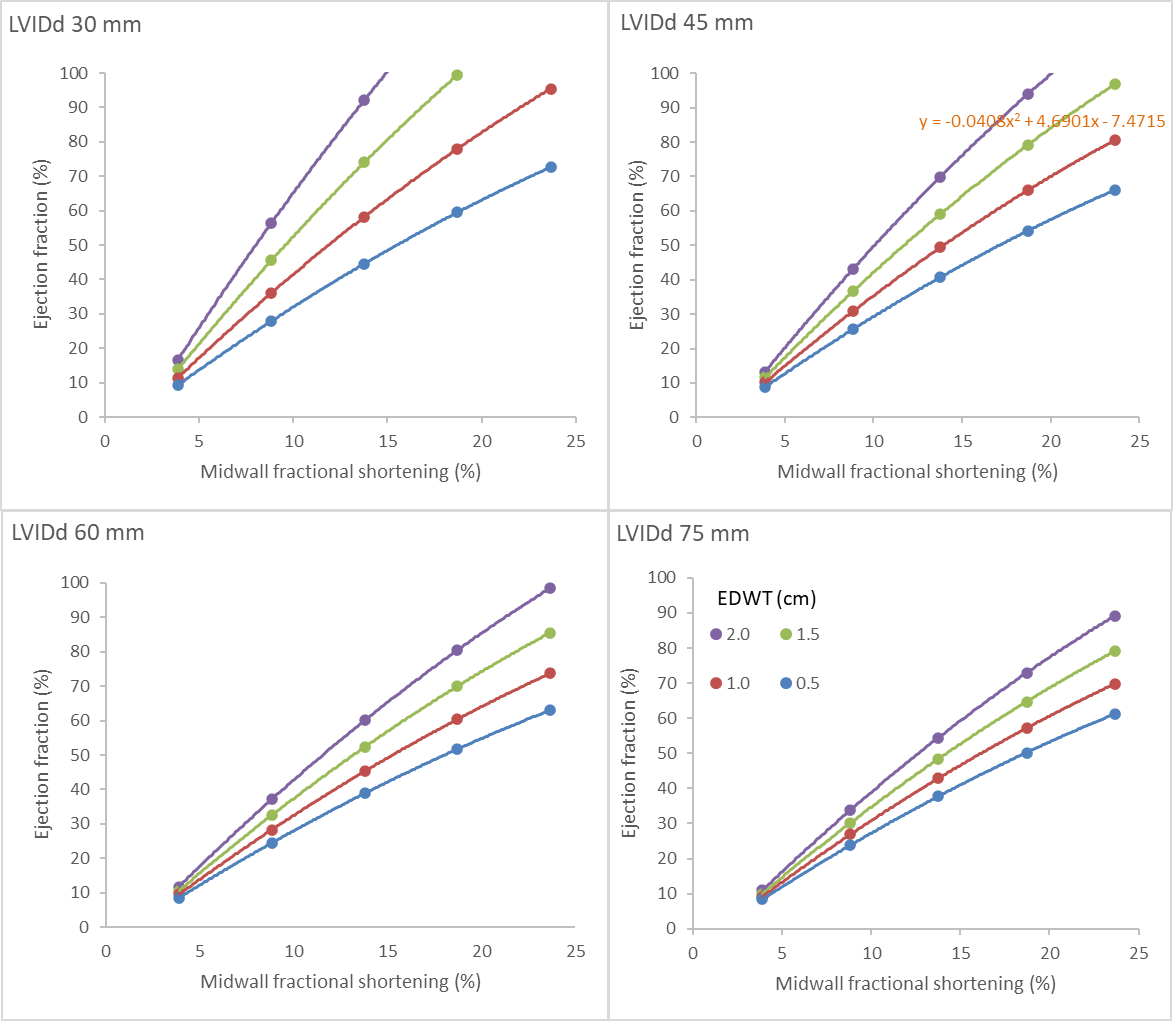


*2. Why is the LVEF higher in females?*

| **Age group** | **Parameter** | **Male** | **Female** | **Difference** | **Calculated†** |
| --- | --- | --- | --- | --- | --- |
| **Young athletes** | LVEF | 59% | 62% | +3.0% | +3.1% |
|  | EDWT (mm) | 8.7 | 7.3 | -1.4 |  |
|  | LVIDd (mm) | 54.8 | 49.2 | -5.6 |  |
| **Veteran athletes** | LVEF | 60% | 66% | +6% | +4.7% |
|  | EDWT (mm) | 9.1 | 8.0 | -0.9 |  |
|  | LVIDd (mm) | 52 | 46 | -6 |  |

The mean values for LVEF, EDWT and LVIDd are shown for each cohort.

†Calculated LVEF assumes an increase in LVEF of 2.6% for every 1 mm increase in EDWT and a 1.2% decrease in LVEF for every 1 mm increase in LVIDd based on mathematical modelling (Table 1).[6]

$$EF\% = 0.97\times{LVIDd}^{2} - 14.7\times LVIDd + 113$$

LVIDd in mm and when EDWT = 10 mm and midwall circumferential strain =-18.7%

$$EF\% = 3.0\times{EDWT}^{2} - 19\times EDWT + 44$$

EDWT in mm and when LVIDd = 45 mm and midwall circumferential strain =-18.7%

*3. How does longitudinal contractance measured by numerically (using stress‒strain curve) vs. analytic method (GLASED equation) compare in normal individuals?*

A typical normal pressure-strain curve was obtained (from Loncaric et al Int J Cardiovasc Imaging 2021 Vol. 37 Issue 1 Pages 145-154) and extracted using Engage digital software. The curve was divided into 12 systole nominal strain intervals (Fig. A). The longitudinal engineering stress was calculated for each strain interval using the Lamé equation to approximate the true stress and true strain using the internal dimensions and wall thickness in diastole and systole from a normal group (from D. H. MacIver, et al Sci Rep 2022;12:1-14) (Fig. B). This study was selected because of the much wider geometric changes than are present in athletes and to include dilated LVs (high stress) with low strain and a non-dilated, thick-walled (low stress) group.

The active strain energy density was calculated for each change in longitudinal strain, and the total strain energy density was obtained via numerical integration using the trapezoidal method, which yielded a longitudinal contractance of 2.80 kJ/m^3^ (Fig. C). The active strain energy is equal to the area under the blue curve in Figure D. Half the area of the rectangle, the peak nominal stress by peak strain (red box) produces a blue triangular area (Fig. D); therefore, equation $\mathrm{GLASED}=\frac{1}{2} \times\sigma_{l} \times\left| \epsilon_{l} \right|$ results in a GLASED of 2.71 kJ/m^3^ (Fig. 4). The approximation for GLASED is achieved because the vertical hatched area is comparable to the horizontal hatched area. See[13] for further details.


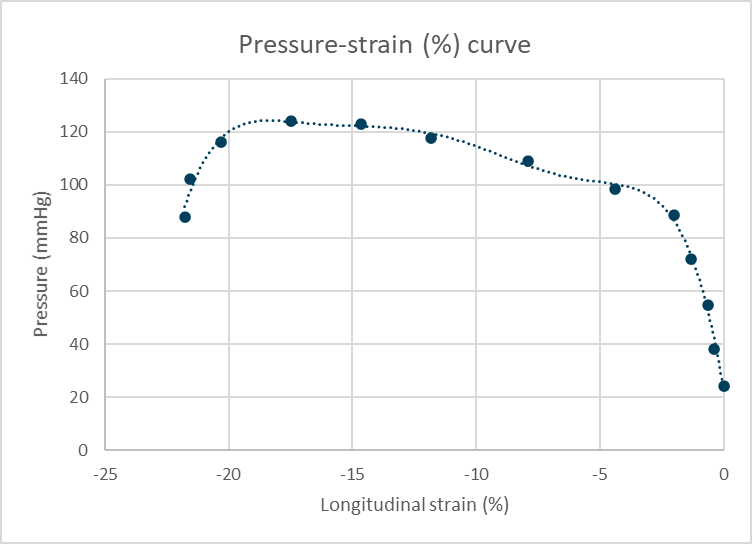

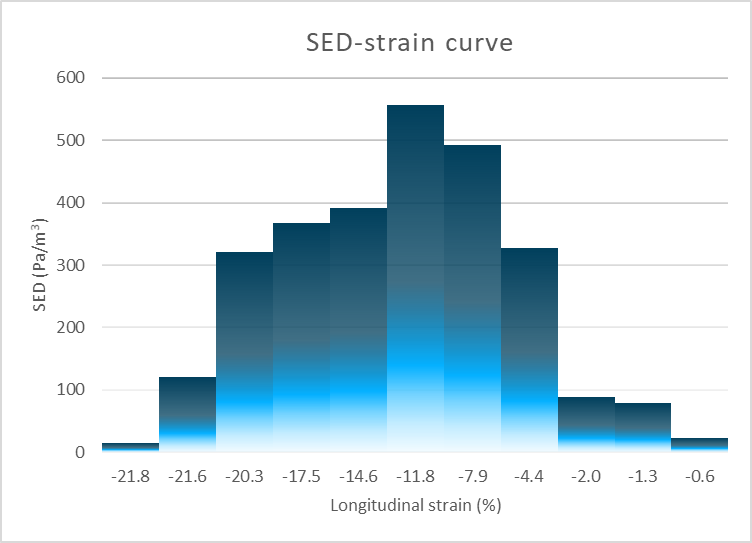

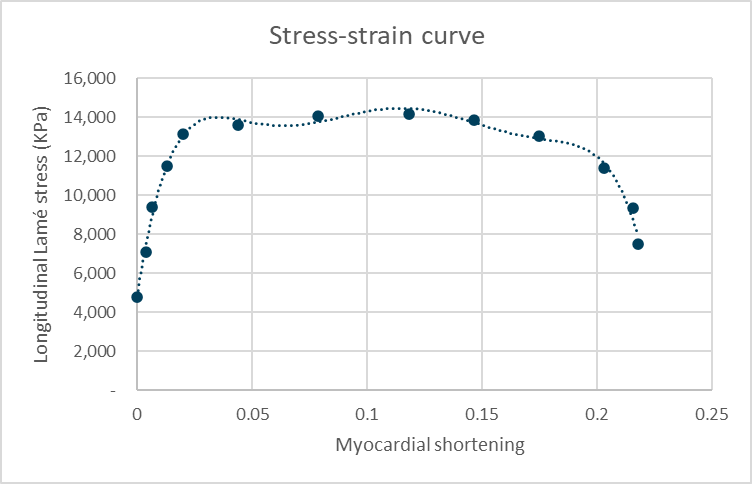

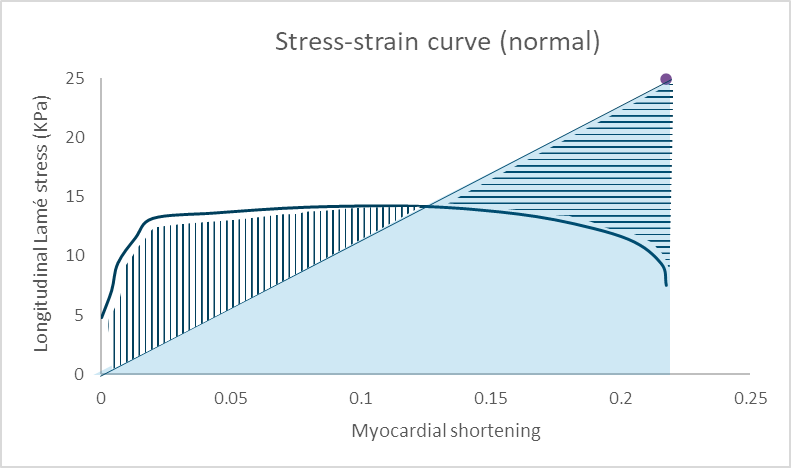


B

C

D

A

*4. How does longitudinal contractance measured numerically using stress‒strain curves vs. the analytic method (GLASED) compare in disease?*

The following data were obtained from “Left ventricular active strain energy density is a promising new measure of systolic function D. H. MacIver, et al Sci Rep 2022;12:1-14”, as this provided a wide range of GLASED results from distinct phenotypes of left ventricular diseases with contrasting geometric abnormalities.

The reference curve was divided into 40 strain points to obtain 39 intervals, and the stress‒strain curves were plotted as described above for the mean values in the normal control, hypertensive cardiomyopathy, dilated cardiomyopathy and cardiac amyloid cohorts. Nominal stresses are shown as crosses, and true stresses are shown as data points.


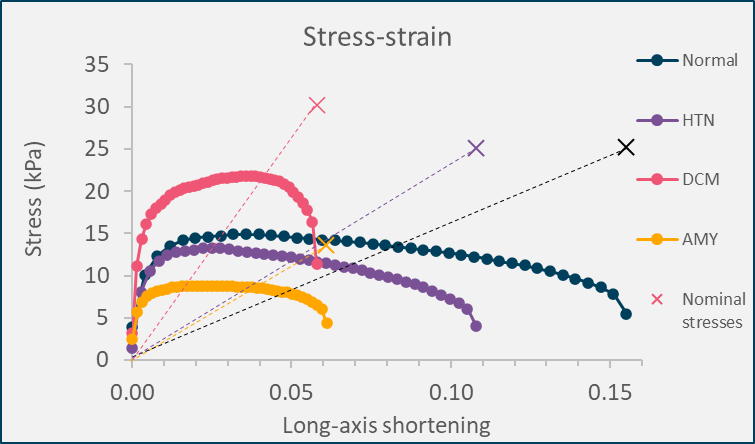


The areas under the curves were obtained using the trapezoidal method (numerical integration), and the GLASED values were obtained using the equation provided above (analytic method).

The following graph was obtained by plotting the two methods.


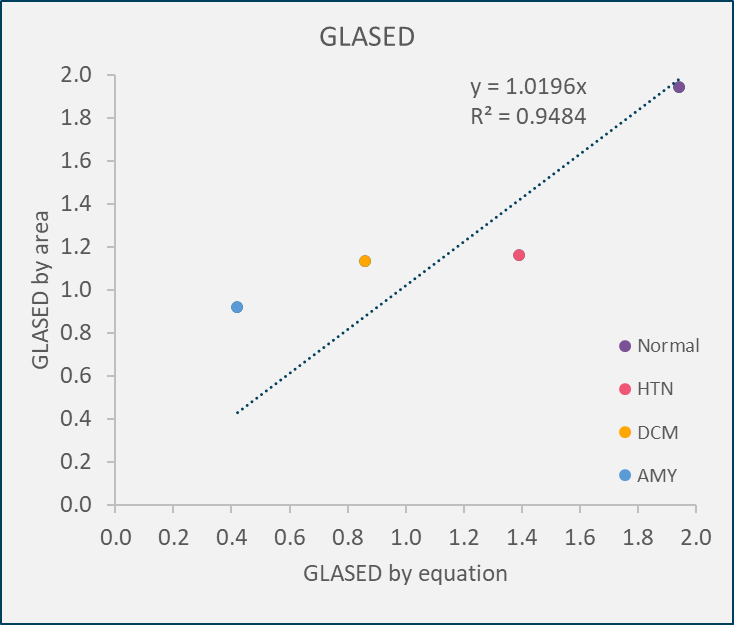

Supplement: Supplementary file 1 — Supplementary Material 1 [file 44156_2024_52_MOESM1_ESM.docx]
